# Evaluation of the implementation and associated effects of advanced access in university family medicine groups: a study protocol

**DOI:** 10.1186/s12875-020-01109-w

**Published:** 2020-02-21

**Authors:** Mylaine Breton, Lara Maillet, Arnaud Duhoux, Sabina Abou Malham, Isabelle Gaboury, Luiza Maria Manceau, Catherine Hudon, Isabel Rodrigues, Jeannie Haggerty, Nassera Touati, Marie-Claude Beaulieu, Christine Loignon, Marie-Thérèse Lussier, Isabelle Vedel, Jalila Jbilou, France Légaré

**Affiliations:** 1 Faculty of Medicine and Health Sciences, Canada Research Chair - Clinical Governance in Primary Health Care, Université de Sherbrooke - Campus Longueuil, 150 Place Charles-Le Moyne, Office 200, Longueuil, QC J4K 0A8 Canada; 2 École Nationale d’Administration Publique, Montreal, QC G1K 9E5 Canada; 3 Faculty of Nursing, Université de Montréal, Montreal, QC H3C 3J7 Canada; 4 Faculty of Medicine, Université de Montréal, Montreal, QC H3C 3J7 Canada; 5 Faculty of Medicine, McGill University, Montreal, QC H3G 2M1 Canada; 6 École de psychologie, Université de Moncton, Moncton, NB E1A 3E9 Canada; 7 Faculty of Medicine, Université Laval, Québec, QC G1V 0A6 Canada

**Keywords:** Primary healthcare, Advanced access, Timely access, Relational continuity, Family medicine, Teaching units, Implementation study, Case study, Organizational change

## Abstract

**Background:**

Timely access in primary health care is one of the key issues facing health systems. Among many interventions developed around the world, advanced access is the most highly recommended intervention designed specifically to improve timely access in primary care settings. Based on greater accessibility linked with patients’ relational continuity and informational continuity with a primary care professional or team, this organizational model aims to ensure that patients obtain access to healthcare services at a time and date convenient for them when needed regardless of urgency of demand. Its implementation requires a major organizational change based on reorganizing the practices of all the administrative staff and health professionals. In recent years, advanced access has largely been implemented in primary care organizations. However, despite its wide dissemination, we observe considerable variation in the implementation of the five guiding principles of this model across organizations, as well as among professionals working within the same organization.

The main objective of this study is to assess the variation in the implementation of the five guiding principles of advanced access in teaching primary healthcare clinics across Quebec and to better understand the influence of the contextual factors on this variation and on outcomes.

**Methods:**

This study will be based on an explanatory sequential design that includes 1) a quantitative survey conducted in 47 teaching primary healthcare clinics, and 2) a multiple case study using mixed data, contrasted cases (*n* = 4), representing various implementation profiles and geographical contexts*.* For each case, semi-structured interviews and focus group will be conducted with professionals and patients. Impact analyses will also be conducted in the four selected clinics using data retrieved from the electronic medical records.

**Discussion:**

This study is important in social and political context marked by accessibility issues to primary care services. This research is highly relevant in a context of massive media coverage on timely access to primary healthcare and a large-scale implementation of advanced access across Quebec. This study will likely generate useful lessons and support evidence-based practices to refine and adapt the advanced access model to ensure successful implementation in various clinical contexts facing different challenges.

## Background

### Importance and impact of the research

**Fig. 1:**
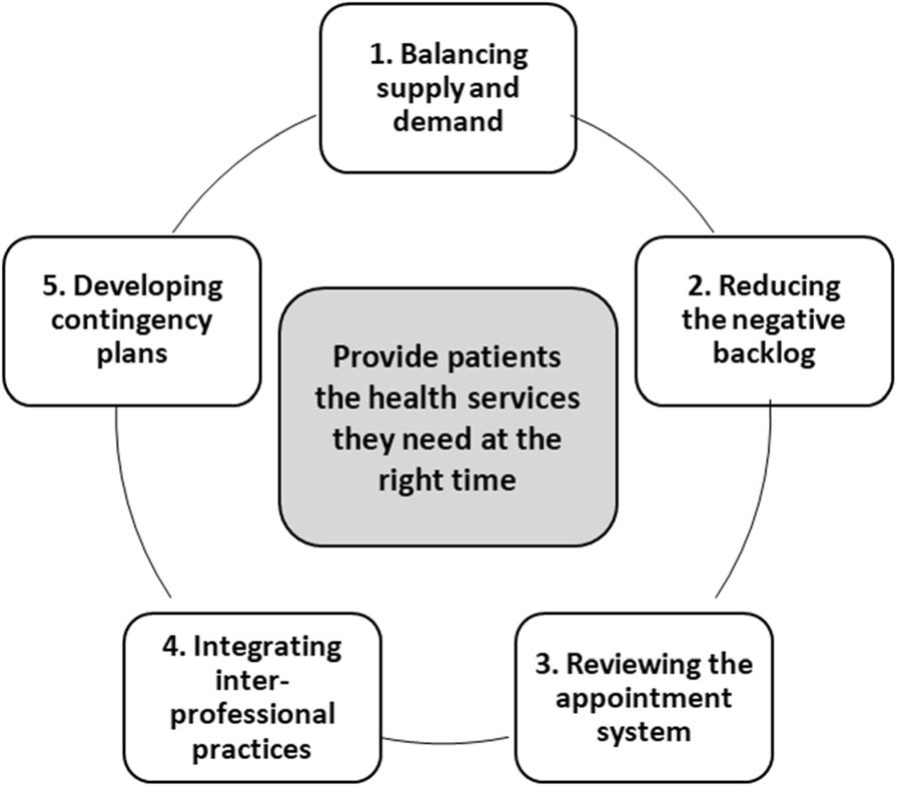
Five guiding principles of Advanced Access model

Accessibility is one of the major concerns facing health systems worldwide [1]. Having access to health services is a high priority for the population, clinicians and decision-makers alike [2]. Timely access, such that patients are able to access care when they need medical attention, often referred as same or next day appointment is one of the 10 pillars of the *Patient-centered Medical Home* [3]. Among many interventions developed around the world to improve timely access, advanced access figures as one of the most highly recommended model to reduce wait times in primary care settings [4–6]. Originally developed in the United States, advanced access has been adopted in many countries and is considered a cornerstone of primary care services of high quality and performance. Based on greater accessibility linked with patients’ relational and informational continuity with a primary care professional or team [7], this organizational model is based on five guiding principles (see Fig. 1) [8]: 1) Balancing supply (appointments available) and demand (requests for appointments) consists of assessing the need for services by weighting the patients’ needs according to their medical conditions and their age and adjusting the supply of services accordingly. 2) Reducing the negative backlog by eliminating the wait list and setting up a communication strategy using a range of tools (e.g. letters, telephone welcome message, notice in the local newspaper) to inform and educate patients about the new model. 3) Reviewing the appointment scheduling system consists of planning physicians’ schedules over a short term (two to three weeks) to be able to anticipate demand for consultations and to permanently offer appointment slots (e.g. same-or next day) for acute and urgent cases. 4) Integrating inter-professional practice requires optimizing the professional roles of other healthcare providers, and consequently redirecting patients to the appropriate provider to respond to patient’s needs in a timely manner. 5) Developing contingency plans facilitates planning for increased demand such as flu season, and proactive planning when providers are absent. Replacement coverage is organized either informally among counterparts or formally at the clinic level, to ensure that the capacity responds to patient demands at all times. Thus, implementing advanced access requires a major organizational change implying more broadly reorganizing the practice of the whole team members (administrative staff and health professionals) to be more patient-focused and efficient [9, 10].

Many studies have been conducted on advanced access in different countries, particularly the United States and the United Kingdom, which have shown positive impacts in terms of reduced wait time for the third available appointment [11–14], reduced missed appointments (no-shows) [13], and increased satisfaction among both professionals [15, 16] and patients [13]. Advanced access is becoming increasingly popular in Canada. However, despite its wide dissemination, a considerable variation in the implementation of the five guiding principles of this model has been observed. These variations in implementation can influence the observed impacts of advanced access, making it difficult to distinguish the impacts attributable to the model itself from those linked to the context and to differences in implementation level [14, 17]. Earlier research has focused primarily on the impacts of advanced access [12–14, 16, 18], and few studies have analyzed the implementation challenges [17, 19–21]. Nevertheless, very few studies to date provide an in-depth understanding of the contextual factors that explain these variations in implementation level, a field that remains largely unexplored.

### Study context

This model has been endorsed by several professional associations across Canada including the College of Family Physicians of Canada. In Quebec, advanced access was first introduced in 2012 and is at present widely promoted by the Quebec College of Family Physicians (CQMF), as well as by the Ministry of Health and Social Services (MHSS). Numerous training sessions have been put in place to support its adoption and dissemination at the provincial level reaching out more than 2000 health care providers and administrative staff (nurses, physicians, and administrative assistants). Over the past 4 years, the majority of family physicians, in close partnership with other professionals working in primary care settings, have implemented advanced access in their organization. We conducted the first two studies (2014, 2016) to understand the early experiences of implementing advanced access with the first adopters family physicians [7], as well as with the first university family medicine groups (UFMGs) who implemented it in Quebec [22]. Results showed not only a wide variation in its implementation levels, but also different combinations of its key guiding principles, among medical practices, as well as among professionals working within the same settings. Data did not allow us to differentiate and understand more deeply the influence of contextual factors on the implementation of the different guiding principles. To our knowledge, no Canadian studies have been conducted since advanced access has been implemented on a more systematic scale, among various primary care organizations. Despite that advanced access has been widely spread, the considerable implementation variations observed suggest disparities in timely access among the population. Thus, there is a need to better understand the underlying reasons for these implementation variations of advanced access and to assess their impact on the intended outcomes. The knowledge generated form this research will be useful to identify context-specific strategies to ensure a successful implementation of advanced access in primary care organizations and ultimately to improve timely access to care.

All UFMGs in Quebec are required to implement an advanced access model based on the five guiding principles. UFMGs are a core primary care model and training sites for all family medicine residents and many other health care professions. UFMGs are an exemplary setting that provides early exposure of future health professionals’ to best practices during their training program and are potentially conducive to implementing advanced access in their future practice [23]. Aside from the exploratory study [24] that we conducted, few studies has been identified on the impact of variations in advanced access implementation in various teaching settings [25]. We observed different models of implementation of advanced access by residents, ranging from a simple pairing/twinning (i.e., sharing a panel of patients between two residents) to the implementation of a joint care model with nurses or a subteam (teamlet) of professionals, in which the resident is included. The results also revealed a variety of implementation challenges faced by the organization itself, and many others faced specifically by residents (e.g., ensuring a balance between timely access and relational continuity of care) that warrant further investigation in larger-scale studies.

### Study objectives

The primary objective of this study is to assess the variation in the implementation of the five guiding principles of advanced access in UFMGs across Quebec and to better understand the influence of the contextual factors on this variation and also on outcomes. The specific research objectives are to:

1. Measure the variability in the implementation of the five guiding principles of advanced access across all UFMGs in Quebec and identify the contextual factors influencing the implementation of advanced access;
2. Gain a deeper understanding of how the contextual, organizational and professional factors influence the implementation of advanced access in four UFMGs;
3. Compare the outcome indicators of advanced access in four UFMGs with regards to its implementation levels (e.g. average wait time for the third appointment, average rate of missed appointments, relational continuity of the team, attendance rate at the UFMG).

### Literature review

Few studies have examined the factors that influence implementation of the guiding principles of advanced access. They have mainly measured the implementation of some guiding principles [17, 26, 27] (e.g., review of the appointments system) while overlooking other principles (e.g., integrating interdisciplinary practice) and the implementation context. Goodall et al. [26] measured the variation in guiding principles in 245 general medicine clinics in England, without considering the contextual factors that influenced their implementation, nor the impact of those variations in implementation levels on outcomes. Conducting further analyses to assess the implementation context and the impact of implementation variations on patients’ experiences was one of their recommendations. Within the same project, Salisbury et al. [28] complemented this analysis by comparing clinics that had implemented advanced access with those that had not, to determine the impact of implementation variations on wait times for obtaining an appointment, continuity of care, appointment availability, and workload. However, their study was limited to assessing the whole advanced access model without distinguishing among the different guiding principles. As part of the same project, Pope et al. [17], in a sample of eight clinics, attempted to understand and explain the variations in implementation levels of the guiding principles and their impact in terms of benefits for patients and reduction in the numbers of missed appointments. Nevertheless, without adopting any comprehensive conceptual framework, they were able to only identify a few factors (misunderstanding or poor knowledge of the advanced access model, confusion between advanced access and same-day access to appointments, external incentives, and informal organizational behaviour) to explain variations in the implementation of advance access. Pickin et al. [27] conducted an assessment among general physicians in Great Britain on the implementation of the guiding principles of advanced access and revealed a number of barriers to its implementation, such as lack of resources and time to make a change, and a resistant culture to changing practice. Only one study, conducted by VanDeusen et al. [10, 29] considered implementation context and attempted to measure variations in implementation of advanced access among different clinical settings and their impacts on patient outcomes. Within an overall assessment of the effectiveness of advanced access implementation on a national scale (United States), the authors measured the extent of implementation of advanced access in 78 primary and specialty care clinics. Adopting a multidimensional conceptual framework, they analyzed three categories of factors (management structure and processes, staff and team capacities, and clinic context in terms of logistics and physical space) associated with the implementation level and their impacts on wait times and patient satisfaction. Their analysis showed that, despite a wide variation in the implementation of the guiding principles across the primary and specialty care clinics, four factors (time devoted to managing change, clinical managerial support, feedback on team performance, level of knowledge/skills) were significant predictors of this implementation. Also, broader implementation of advanced access was associated with a statistically significant improvement in patient access (shorter wait times for clinic appointments in primary care, orthopedics, and urology) and a higher patient satisfaction [10].^.^ Their results highlight the importance of understanding the dynamics and the role of context in successful implementation [30].

## Methods

### Conceptual framework

**Fig. 2:**
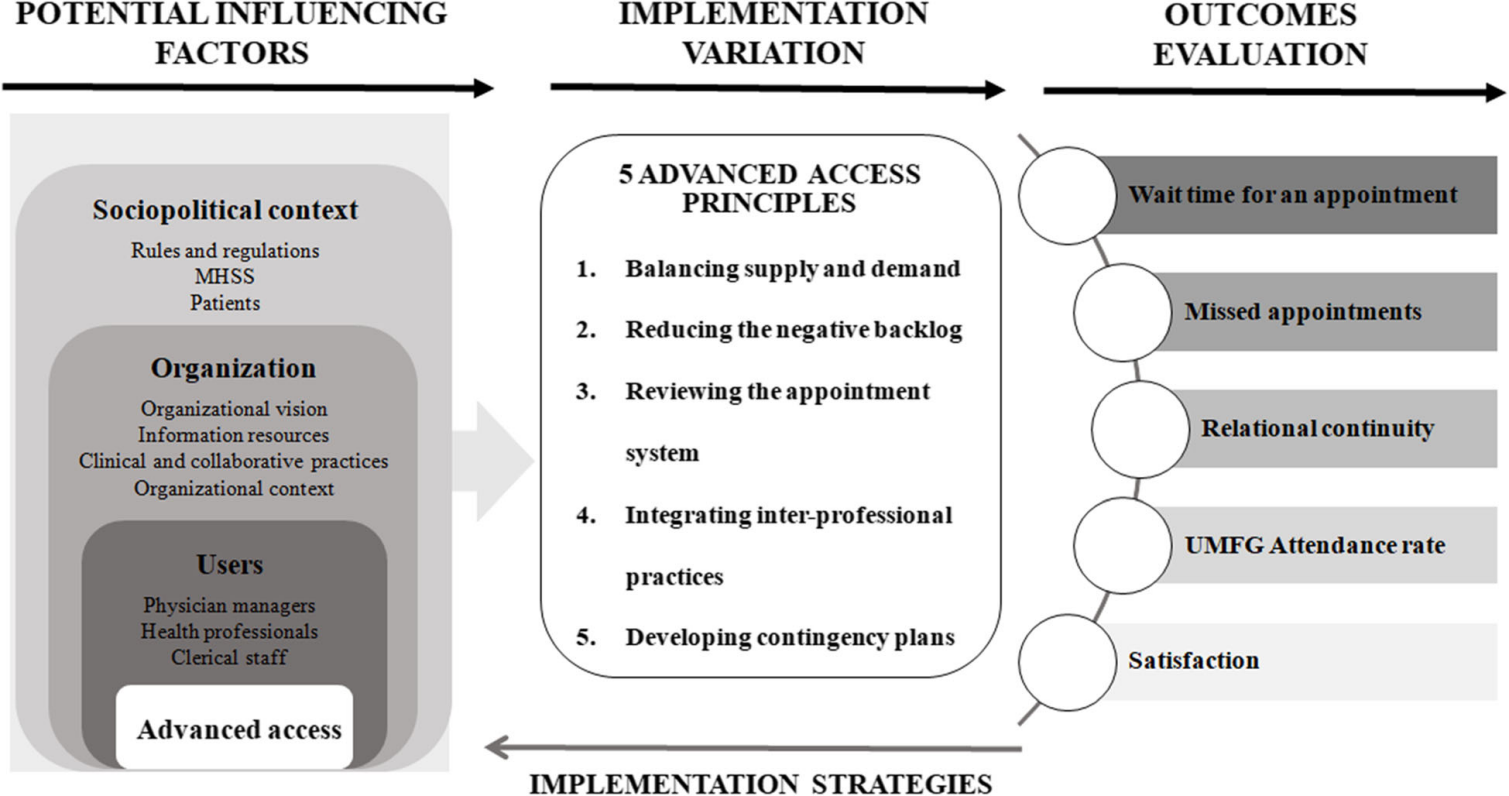
Conceptual framework

The conceptual framework for this project builds primarily on the multilevel framework of Fleuren et al. [31], which presents different factors that could potentially influence the implementation of an innovation (see Fig. 2). Our framework considers various factors -sociopolitical, organizational, professional, related to advanced access that could influence the implementation effectiveness and impact, of advanced access (shorter wait times for appointments, reduced missed appointments, and increased relational continuity, attendance at the UFMG, and patient satisfaction). Ultimately, identifying the influential factors will help to adapt the implementation strategy of advanced access to the local context [31] and achieve the intended outcomes. Thus, *sociopolitical* factors refer to the sociocultural and political environment and to the characteristics of patients living in the community. ***Organizational*** factors relate to the primary care organizations in which the intervention is implemented, particularly in terms of coordination, organizational policies, and financial, human, and material resources. ***Professional*** factors refer to characteristics of those working in the organization and interacting with patients such as their field of practice and expertise (e.g., qualifications) [32], level of knowledge, willingness to comply, and sense of self-efficacy in conducting activities within implementation process, etc. They also incorporate the standards and values that play a significant role in the implementation process [33]. Factors related to the advanced access model include procedural clarity, observability of results, compatibility, completeness, and complexity.

### Methods

**Fig. 3:**
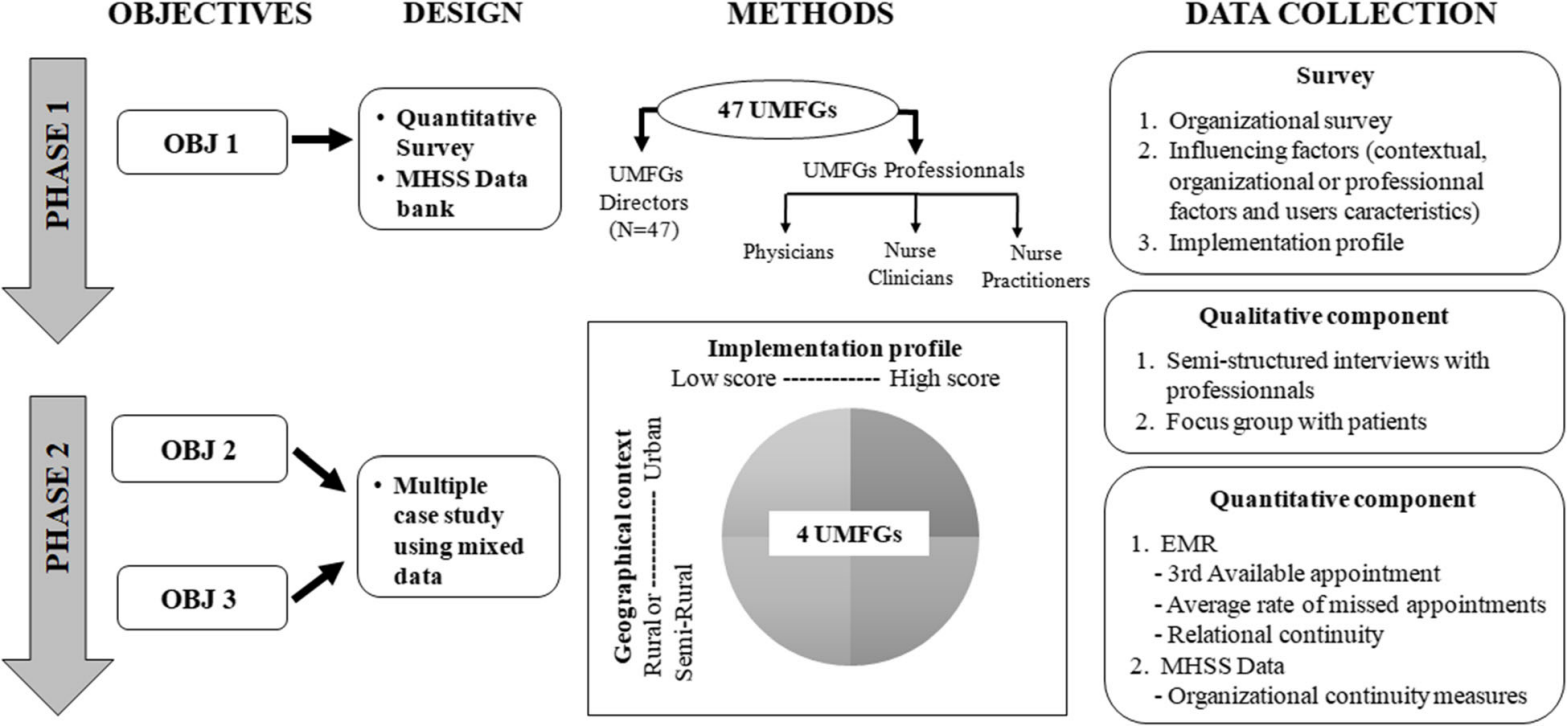
Methods

This study will be based on an explanatory sequential design that includes phase 1:a ***quantitative survey*** (objective 1) conducted in all the UFMGs, and phase 2: a ***multiple case study*** (objectives 2 and 3) using mixed data [34]. Phase 1 will allow us to develop a comprehensive profile of the implementation levels of the five guiding principles of advanced access across the province; to assess the intra (among types of professionals, namely the physicians, nurses, and primary care nurse practitioners), and inter (across all UFMGs) implementation variability; and to analyze the factors influencing the implementation variation of advanced access within teaching organizations. Phase 2 will provide an in-depth analysis and explanation of the influence of the different factors on implementation, to assess the influence from the perspectives of key users of the model (professionals and patients); and lastly will enable the measurement of the intended outcomes of advanced access. The qualitative and quantitative components are thus complementary *(*see Fig. 3*)*.

#### Step 1: Survey of all UFMG directors and the professionals working in the UFMGs

##### Data sources

Three data sources will be used:

1. The **Minister of Health and Social Services (MHSS) data bank on UFMGs**, which includes a range of data, in particular the number of patients registered and the number and types of different professionals.
2. An **organizational survey** of the 47 directors of all UFMGs.
3. **Providers’ survey** covering three types of professionals working in the UFMGs.

##### Measurement instruments

The *implementation of advanced access* will be assessed in both the organizational and provider surveys by measuring each of the five guiding principles of advanced access [35, 36] including their different sub-dimensions using a 5-point Likert scale. This section of the questionnaires is based on the work of: 1) VanDeusen et al. [10], who developed a questionnaire reflecting the operationalization of certain sub-dimensions of the guiding principles, as well as on a practice guide, *Advanced Access and Efficiency Workbook for Primary Care*, developed by *Health Quality Ontario* [37], which presents the operational definitions of the guiding principles of advanced access and 2) Orchard, King, Khalili et Bezzina [38] who designed and validated a short version questionnaire entitled *Assessment of Interprofessional Team Collaboration Scale*, particularly eight items on partnership. The *MIDI-Measurement Instrument for Determinants of Innovation*, developed by Fleuren et al. [31] will be used in both surveys to assess the *factors influencing the implementation of advanced access* in the UFMGs. The version of UFMG directors also includes an organizational survey based on the work of the Canadian Institute for Health Information [39], whose approval is already obtained. Some questions were, however, removed given that they were considered less relevant for this study or when the information was already available in the MHSS data bank. A pretest was done in the spring 2019 within a pilot project conducted in three UFMGs in one region (Estrie). Funded by the *Fondation Vitae*, this pilot project had enabled us to test the questionnaires in a real-world context and to improve them for the present study.

##### Participant recruitment

All UFMGs (*n* = 47) in the province will be invited to participate. To encourage their participation, we will adopt a personalized approach via one of the four practice-based research networks on primary care (PBRNs). We will request the support of the directors of the departments of medicine and emergency medicine of Quebec’s four faculties of medicine, along with four physician members of our team involved in each of the PBRNs. The 47 UFMG directors will be invited by email to respond to the organizational survey which will be developed in a digital format using the *Survey Monkey* platform. Based on the pilot survey, we are confident we will be able to achieve a response rate of about 80% from the UFMG directors (organizational survey), given the close partnerships already established with clinical leaders involved in this project, as well as the previous experience of our research team members who have conducted projects in UFMGs that generated high satisfactory response rates.

With respect to the questionnaires for professionals, all physicians, residents, nurse clinicians, and primary care nurse practitioners working in the UFMGs will be invited by email to participate in the study. Working in close partnership with the PBRNs, this study will be promoted, and the invitations will be sent by UFMGs key collaborators (e.g. manager, physician in charge or head of research, continuous quality improvement officer).

Three reminder emails will be set-up and sent at regular intervals, i.e., after 1, 2, and 4 weeks, to increase the response rate [40]. Currently, there are approximately 980 physicians, 80 nurse clinicians, and 90 PCNPs working in the UFMGs. To reach a power of at least 94.9%, we estimate that we will need a response rate of 25% for physicians and residents, with a 5% margin of error. To achieve a power of at least 82%, we estimate that we will need a response rate of 40% for the nurses and primary care nurse practitioners.

##### Analysis

To respond to *objective 1* (assess the implementation of the five guiding principles of advanced access in the UFMGs), a score will be calculated for each principle, by type of respondent. Implementation scores will be assessed for each advanced access guiding principle by type of professional. Then, scores will be aggregated to produce group scores at the UFMG level for each of the above-mentioned principles. The median or average of all UFMGs’ score will be used as a threshold to determine the implementation categorization (e.g., strong/weak) of the advanced access for each principle. Descriptive analyses in terms of proportions, means, or standard deviations will be generated to determine the implementation scores of each principle and the factors influencing implementation. Multiple linear and logistic regression analyses will be performed using SAS 9.4 to determine the influence of different factors on implementation scores.

The Phase 1 results will be used to produce an implementation profile (e.g., strong/weak) of the UFMGs to guide the selection of the four cases which will be further analyzed in depth in Step 2.

#### Step 2: Multiple contrasted case study based on mixed data

##### **A)** Qualitative component

***Data collection***

We will select four contrasted cases representing implementation profiles (*n* = 2 cases with high score, n = 2 cases with low score) and various geographical contexts and organizational size of UFMG (n = 2 cases in urban areas, n = 2 cases in rural/semi-rural areas). Previous studies have shown that context have a major influence on the types of collaborative practices among professionals [41].

For each UFMG in the study (*n* = 4), we will first perform a documentary analysis (e.g., algorithm of the patient’s path through advanced access, operating rules for team members). Then, a researcher and the research coordinator will conduct and record semi-structured interviews (60 min; at the UMFG) to reach data saturation (*n* = 11 interviews/case: two physicians, two PCNPs, two nurse clinicians, the physician in charge, one manager, two administrative assistants, one continuous quality improvement officer). The professionals will be recruited by email using purposive and snowball sampling strategies addressing first the medical director [42].

In addition, patients’ perceptions are key to the process of adopting the advanced access model and to its successful implementation. In each of the four UFMGs under study, a researcher and the research coordinator will conduct and record a focus group of patients (120 min; at the UFMG) with different characteristics (age, sex, education level, comorbidity) (*n* = 8–10 patients/focus group) [43] to assess patients’ experiences of care in relation to accessibility issues. This type of sample will be useful to represent and describe the internal diversity of the UFMG’s group of patients. Patients will be recruited in each of the selected UFMGs using different strategies: a poster will be displayed in the waiting rooms; the administrative assistants will be informed of the project and encouraged interested patients in research to participate in the study. We will provide them with letters of invitations containing the required information that patients will need if interested to contact the researchers. Lastly, the professionals themselves could provide verbal information on the research project to patients during their visits. These techniques have already been used in our previous project [7, 22] on advanced access and were successful in recruiting and conducting three focus groups in a single UFMG. The patient partners on our advisory team will be involved in the data collection and results interpretation.

***Analysis***

Thematic analysis based on our conceptual framework will be used to analyze the semi-structured interviews and focus groups. The research coordinator will code the interviews with NVivo software using a mixed deductive (conceptual model) and inductive strategy. A summary list of initial codes based on our conceptual model (five guiding principles of advanced access; contextual, organizational, and professional factors; etc.) will serve a priori as a coding grid. It will be modified and enhanced as the analyses progress. Coding will be controlled using a double coding technique conducted by the research coordinator and a researcher. Parallel and independent coding will be done for the first five interviews, after which the results will be compared. This process will be repeated until a consensual list of initial codes is obtained as well as an inter-coder fidelity greater than 90% [44].

##### B) Quantitative component

***Data collection***

Impact analyses will be conducted in the four selected UFMGs. We will use data retrieved from the electronic medical records (EMR) for the three first indicators presented below and data from the MHSS data bank for the fourth indicator. All UFMGs in Quebec have a functional EMR system. Because the UFMGs are attached to health facilities, we will be able to obtain all the EMR data for research purposes with the authorization of the directors of professional and hospital services without needing explicit consent from patients. On average, there are 20 physicians, 18 residents, two nurse clinicians, and one primary care nurse practitioner working in each UFMG, with an average of 5112 patients registered [45]. Thus, we calculated the estimated numbers in our samples as follows: around 160 professionals and 20,000 patients. The four indicators will be measured monthly over a one-year period.

1. *The average wait for the third available appointment.* This is the most accurate indicator for measuring the impact of advanced access implementation [37, 46]. It is used to assess the time required for an appointment and refers to the number of business days before the third available appointment in a professional’s schedule, for a regular or follow-up appointment [47]. It reflects availability more accurately than using the first or second available appointment, as these could result from a recent cancellation or an unanticipated event [13, 27]. As suggested by several authors, including the *Quality Improvement Guide* [48], Pickin et al. (2004) [27], and Jones et al. (2003) [49], we will calculate, every Tuesday, the median number of days until the third available appointment, and then the monthly average for each professional in the UFMGs [27].
2. *Average rate of missed appointments (no-shows).* An underlying premises of advanced access is that providing a system of timely appointments leads to greater efficiency, with fewer missed (no-show) appointments [37]. Several studies have shown positive impacts on this indicator [50]. No-shows are defined as appointments that are scheduled but not used, excluding those cancelled or changed by the professional, and that are registered by the administrative staff in the EMR. We will calculate on a monthly basis, for each professional, the number of no-shows in relation to the total number of scheduled appointments [51]*.*
3. *Relational continuity.* Relational continuity is an important feature of primary care quality [48]. Patients followed by a professional whom they trust generate fewer visits [48]. While new organizational models rely on patients being managed by a team of professionals, advanced access aims first to optimize appointments with the professional to whom the patient is affiliated, depending on the need for the visit and the professional’s availability. Relational continuity is calculated as the number of visits with the physician and other team partner (e.g., nurses, residents) responsible for that patient’s follow-up care divided by the total number of that patient’s visits at the clinic [37*,*^48^]*. T*his indicator can be broken down into two complementary measures: 1) the number of visits the patient had with his/her affiliated physician divided by total number of visits to the UFMG; and 2) the number of visits with other team partner following that patient (e.g., nurses, residents) divided by the total number of visits to the UFMG. Relational continuity with the team will be obtained on a monthly basis for each patient from the sum of these two measures.
4. *Organizational continuity measures*. The aim of advanced access is that patients will consult the primary care clinic to which they are affiliated to satisfy the majority of their needs. Organizational continuity measures the proportion of primary-care medical services patients receive at their UFMG where they are attached. This is measured by the number of consultations at the UFMG divided by the total number of primary care consultations (including visits both to the UFMG and to the emergency room for less urgent cases). Monthly measures of attendance rates of UFMG patients will be obtained from the MHSS, a partner in this study, which holds accurate measurement data of this indicator for all FMGs and UFMGs since January 2016.

***Analysis***

Descriptive analyses will be performed for all data, as well as graphic representations of the changes in monthly rates for each indicator aggregated to the level of each of the four selected UFMGs. Given the hierarchical nature of the data (repeated measures nested at the patient [indicator 3] or professional [indicators 1 and 2] level, which in turn are nested within the UFMGs), we will use multilevel analyses. These models will be adjusted to examine the effects of the various factors and of the implementation levels of advanced access on each of the three indicators. This type of model allows us to take into account the correlation between repeated measures for a single individual (patient or professional) over the study period, as well as missing data and patients lost to follow-up [52]. For the 4th indicator, trends will be analyzed using *join point* regression analyses [53]. This technique will be used to compare attendance rates among the 47 UFMGs and detect whether there have been significant changes in the direction or size of the linear trends for the rates over the 12 months of the study.

### Integrating the qualitative and quantitative components

We will begin by performing an intra-case analysis. We will analyze the coded material from different data sources (documents, professionals’ perceptions, patients’ perceptions), as well as the impact variables measured for objective 3 and the assessment of patients’ perceptions. Then we will summarize our results in tables and matrices [44]. The matrices will present the results by grouping the codes according to the different themes proposed in our conceptual framework, as well as new themes that emerge during the analysis. Based on the tables and matrices for each case, we will perform a “network thematic analysis” to identify relationships among the various dominant themes (*organizing themes*) and their defining characteristics (*basic themes*) using graphic representation [54]. This analytical approach will facilitate our understanding of the relationships among the different factors and observed effects. We will then perform a cross-sectional inter-case analysis, which involves developing a comparative summary matrix identifying models of similarities and differences among the four study cases, from which we will draw key lessons and recommendations. This analysis approach will help us develop a set of recommendations on all factors influencing the implementation of advanced access and its impacts.

The complementary use of qualitative and quantitative methods will allow triangulation and confirmation of findings and the overall validity of results.

### Dissemination of findings

#### Integrated knowledge application approach

Using a collaborative and partnership-based research approach will ensure that our partners are involved in many deliberative discussions at all stages of the study and will help improving application of the results and to develop strategies for sharing the results with different audiences. Our research team includes several decision-makers and clinicians with the capacity to influence health services organization in Quebec. These people have significant strategic roles and will be key levers for disseminating research results to various audiences at different levels of government. Thus, we will use the dissemination channels available through our partners (MHSS, Quebec Federation of General Practitioners, Quebec Medical Association, CQMF, Quebec Order of Nurses, *Institut National d’Excellence en Santé et en Services Sociaux,* four family medicine departments, Quebec Association of Specialized Nurse Practitioner, Réseau-1, Quebec SPOR-SUPPORT Unit, *Institut Universitaire de Première Ligne en Santé et Services Sociaux*) and the events they will organize. Throughout the research project, information will be shared in the monthly newsletter written by the senior officer for continuous quality improvement for Quebec UFMGs, to which all UFMG directors, managers, and quality leaders subscribe. The research team will also be supported by the Quebec SPOR-SUPPORT Unit to implement innovative knowledge dissemination strategies.

#### Support for knowledge application in practice settings

An interactive face-to-face meeting will be organized with members of each of the four study UFMGs. At these meetings, we will: 1) present the results in a personalized way and discuss the factors that could potentially explain the results obtained; 2) discuss the lessons learned from our study; and 3) identify with them areas for improvement adapted to their implementation context. These discussion meetings will be opportunities to expand our respective knowledge, as well as learning opportunities for the practice settings. The lessons learned from these meetings will be incorporated into the practice guide for implementing advanced access in university settings that we intend to develop and diffuse.

A one-day research symposium will be organized at the end of the project (2022) at the Université de Sherbrooke, Longueuil campus. All the research team members will be invited (researchers, clinicians, decision-makers, patient partners), as well as key stakeholders in the healthcare network and students. We also plan to invite two speakers (Canadian and international) to benefit from their experience and to discuss issues of transferability.

#### Pan-Canadian dissemination

In close partnership with the Family Medicine Department Directors in Quebec and the board of Professional Development and Practice support Division of the College of Family Physician of Canada, we will organize a symposium on the key results of the study in 2021 at the Family Medicine Forum, the largest gathering of family physicians in Canada.

### Potential challenges and mitigation strategies

Three major issues deserve attention. The first has to do with conducting a sequential project. The three co-PIs and the research coordinator will meet regularly to ensure ongoing monitoring of the project. The second is related to the questionnaire response rates. Strategies have been put in place to encourage participation, including a personalized approach by PBRN and accreditation of training for physicians and nurses. There may also be a selection bias [55] which will be taken into account in the statistical analyses. First, during the data collection process, we will follow up on respondents closely to develop customized strategies with the PBRNs to encourage professionals’ participation in the survey. Then, if there is variation in the representativeness of respondents, weighted analyses will be carried out. Finally, for the analysis of qualitative data based on a mixed deductive (conceptual model) and inductive strategy, an inter-rater agreement process will be conducted for the first analyses until a list of initial consensual codes and an inter-coder fidelity is obtained.

## Discussion

This study addresses an important need identified by key stakeholders on timely access. In Canada, only 43% of the population report that they can see a doctor or nurse the same day or the next day when needed. This result is the lowest of all 11 Commonwealth Fund countries [56]. In recent years, advanced access has been widely implemented in primary care organizations as a promising solution to reduce wait times. Its implementation requires a major organizational change based on redesigning the work process of all the administrative staff and health professionals. However, despite its wide dissemination, we observe considerable variation in the implementation of the five guiding principles of this model not only among organizations, but also among professionals working within the same organization. Several scientific articles have studied the impacts of advanced access, and some have analyzed its implementation. Nevertheless, very few studies provide an in-depth understanding of the factors that explain the variations of the implementation levels and their impact on outcomes. This study will make a novel contribution to the field of implementation science and will fill an important gap in the literature on implementing advanced access in teaching primary healthcare clinics.

The findings will enhance understanding of how and why some primary care settings ensure successful implementation of advanced access and reduce wait times while others present a gap of implementation and fail to satisfy their patients’ needs and preferences with regards to timely access. More specifically, they will help to clarify which specific component (or guiding principle) of this complex innovation or which combination of guiding principles are critical to implementation effectiveness and should be prioritized by key stakeholders in particular contexts and by decisions makers to reduce wait times.

Identifying factors associated with successful implementation and positive outcomes provides useful lessons for future implementation and diffusion of advanced access in Quebec and across all Canadian provinces.

## Data Availability

Not applicable.

## Abbreviations

CQMF: Quebec College of Family Physicians
DOSPLI: Department of Integrated Primary Care Services Organization
EMR: Electronic Medical Records
MHSS: Ministry of Health and Social Services
MIDI: Measurement Instrument for Determinants of Innovation
PCNP: Primary Care Nurse Practitioner
RRAPPL: Research Networks on Primary Care Practice
UFMG: University Family Medicine Group

## References

[1] The College of Family Physicians of Canada. Best advice – timely access to appointments in family practice. 2012. http://www.cfpc.ca/uploadedFiles/Health_Policy/_PDFs/2012_Final_Best_Advice_Enhancing_Timely_Access.pdf. Accessed 8 Sep 2018.

[2] Boivin A, Lehoux P, Lacombe R, Lacasse A, Burgers J, Grol R (2011). Target for improvement: a cluster randomised trial of public involvement in quality-indicator prioritisation (intervention development and study protocol). Implement Sci.

[3] The College of Family Physicians of Canada. A vision for Canada: family practice – the patient’s medical home. Position paper. 2011. https://www.cfpc.ca/projectassets/templates/resource.aspx?id=3753&langType=4105. Accessed 8 Sep 2018.

[4] Chapman J, Zechel A, Carter Y, Abbot S (2004). Systematic review of recent innovations in service provision to improve access to primary care. Br J Gen Pract.

[5] Comité Accessibilité – Département de médecine de famille et de médecine d’urgence. Annual Report. Université de Sherbrooke. 2017. https://www.usherbrooke.ca/dep-medecine/fileadmin/sites/dep-medecine/images/RapAn2017/CHUS_rapportannuel_final_HR_6a.pdf . Accessed 8 Sep 2018.

[6] Ansell D, Crispo JAG, Simard B, Bjerre LM (2017). Interventions to reduce wait times for primary care appointments: a systematic review. BMC Health Services Res.

[7] Breton M, Maillet L, Paré I, Abou Malham S, Touati N (2016). Perceptions of the first family physicians to adopt advanced access in the province of Quebec. Canada Int J Health Plann Manage.

[8] Murray M, Berwick DM (2003). Advanced access: reducing waiting and delays in primary care. JAMA..

[9] Murray M (2005). Answers to your questions about same-day scheduling. Fam Pract Manag.

[10] VanDeusen Lukas C, Meterko M, Mohre D, Nealon Seibert M. The Implementation and Effectiveness of Advanced Clinic Access HSR&D Management Decision and Research Center Office of Research and Development Department of Veterans Affairs 2004. https://www.researchgate.net/publication/228478818_The_Implementation_and_Effectiveness_of_Advanced_Clinic_Access. Accessed 8 Sep 2018.

[11] Bennett KJ, Baxley EG (2009). The effect of a carve-out advanced access scheduling system on no-show rates. Fam Med.

[12] Belardi FG, Weir S, Craig FW (2004). A controlled trial of an advanced access appointment system in a residency family medicine center. Fam Med.

[13] Bundy DG, Randolph GD, Murray M, Anderson J, Margolis PA (2005). Open access in primary care: results of a North Carolina pilot project. Pediatrics..

[14] Rose KD, Ross JS, Horwitz LI (2011). Advanced access scheduling outcomes: a systematic review. Arch Intern Med.

[15] Ahluwalia S, Offredy M (2005). A qualitative study of the impact of the implementation of advanced access in primary healthcare on the working lives of general practice staff. BMC Fam Pract.

[16] Hudec JC, MacDougall S, Rankin E (2010). Advanced access appointments: effects on family physician satisfaction, physicians' office income, and emergency department use. Can Fam Physician.

[17] Pope C, Banks J, Salisbury C, Lattimer V (2008). Improving access to primary care: eight case studies of introducing Advanced Access in England. J Health Serv Res Policy.

[18] Fournier J, Heale R, Rietze LL (2012). I can't wait: advanced access decreases wait times in primary healthcare. Healthc Q.

[19] Pierdon S, Charles T, McKinley K, Myers L (2004). Implementing advanced access in a group practice network. Fam Pract Manage.

[20] Mehrotra A, Keehl-Markowitz L, Ayanian JZ (2008). Implementing open-access scheduling of visits in primary care practices: a cautionary tale. Ann Intern Med.

[21] True G, Butler AE, Lamparska BG, Lempa ML, Shea JA, Asch DA (2013). Open access in the patient-centered medical home: lessons from the Veterans Health Administration. J Gen Intern Med.

[22] Abou Malham STN, Maillet L, Gaboury I, Loignon C, Breton M. What are the factors influencing implementation of advanced access in family medicine units? A cross-case comparison of four early adopters in Quebec. Int J Family Med. 2017;1595406. 10.1155/2017/1595406 PMC5523347 28775899

[23] Ministère de la Santé et des Services sociaux. Cadre de gestion des groupes de médecines de familles universitaires (GMF-U). QC: Gouvernement du Québec, 2016. http://publications.msss.gouv.qc.ca/msss/document-001771/. Accessed 8 Sep 2018.

[24] Abou Malham S, Touati N, Maillet L, Breton M (2018). The challenges of implementing advanced access for residents in family medicine in Quebec. Do promising strategies exist?. Med Educ Online.

[25] Hudon C, Luc M, Beaulieu M-C, Breton M, Boulianne I, Champagne L (2019). Implementing advanced access to primary care in an academic family medicine network. Participatory action research. CFP..

[26] Goodall S, Montgomery A, Banks J, Salisbury C, Sampson F, Pickin M (2006). Implementation of Advanced Access in general practice: postal survey of practices. Br J Gen Pract.

[27] Pickin M, O'Cathain A, Sampson FC, Dixon S (2004). Evaluation of advanced access in the national primary care collaborative. Br J Gen Pract.

[28] Salisbury C, Montgomery AA, Simons L, Sampson F, Edwards S, Baxter H (2007). Impact of Advanced Access on access, workload, and continuity: controlled before-and-after and simulated-patient study. Br J Gen Pract.

[29] Lukas CV, Meterko MM, Mohr D, Seibert MN, Parlier R, Levesque O (2008). Implementation of a clinical innovation: the case of advanced clinic access in the Department of Veterans Affairs. J Ambul Care Manage.

[30] Durlak JA, DuPre EP (2008). Implementation matters: a review of research on the influence of implementation on program outcomes and the factors affecting implementation. Am J Community Psychol.

[31] Fleuren MA, Paulussen TG, Van Dommelen P, Van Buuren S (2014). Towards a measurement instrument for determinants of innovations. Int J Qual Health Care.

[32] Greenhalgh T, Robert G, Macfarlane F, Bate P, Kyriakidou O (2004). Diffusion of innovations in service organizations: systematic review and recommendations. Milbank Q.

[33] Damschroder LJ, Aron DC, Keith RE, Kirsh SR, Alexander JA, Lowery JC (2009). Fostering implementation of health services research findings into practice: a consolidated framework for advancing implementation science. Implement Sci.

[34] Creswell JW (2013). Research design: qualitative, quantitative, and mixed methods approaches.

[35] Murray M, Tantau C (1999). Redefining open access to primary care. Manag Care Q.

[36] Murray M, Tantau C (2000). Same-day appointments: exploding the access paradigm. Fam Pract Manag.

[37] Health Quality Ontario. Advanced access and efficiency workbook for primary care. Toronto: Queen’s Printer for Ontario; 2012. www.hqontario.ca/Portals/0/.../qi-aae-interactive-workbook-en.pdf. Accessed 7 Oct 2016.

[38] Orchard C, Pederson LL, Read E, Mahler C, Laschinger H (2018). Assessment of Interprofessional Team Collaboration Scale (AITCS): Further Testing and Instrument Revision. J Contin Educ Heal Prof.

[39] Canadian Institute for Health Information. About the primary health care practice-based surveys. April 2013. https://www.cihi.ca/en/info_phc_handout_en.pdf. Accessed 8 Sep 2018.

[40] Dillman DA, Smyth JD, Christian LM (2009). Internet, mail, and mixed-mode surveys: the tailored design method.

[41] Lamarche P, Maillet L (2016). The performance of primary health care organizations depends on interdependences with the local environment. J Health Organ Manag.

[42] Patton MQ (2002). Qualitative research & evaluation methods.

[43] Poupart J, Deslauriers JP, Groulx LH, Laperrière A, Mayer P, Pires AP (1997). La recherche qualitative: enjeux épistémologiques et méthodologiques.

[44] Miles MB, Huberman AM (1994). Qualitative data analysis: an expanded sourcebook.

[45] RRAPPL U de Montréal – RRSPUM (2015). Utilisation des DMÉ à travers le Réseau-1 Québec.

[46] Mark Murray & Associates. Measurement guide for reporting: advanced access and new patients; 2014. www.wrha.mb.ca/staff/familyphysicians/files/MeasurementGuideforReportingAA.pdf. Accessed 8 Sep 2018.

[47] British Columbia Medical Association. Office practice redesign in primary health care: advanced azccess and office efficiency workbook. BC Medical Association & BC Ministry of Health, Practice Support Program; 2013. http://www.gpscbc.ca/sites/default/files/AAOE%20Workbook_final-REVISED-May%2029-2013_0.pdf.

[48] Ontario Health Quality Council (2009). Quality improvement guide.

[49] Jones W, Elwyn G, Edwards P, Edwards A, Emmerson M, Hibbs R (2003). Measuring access to primary care appointments: a review of methods. BMC Fam Pract.

[50] Steinbauer JR, Korell K, Erdin J, Spann SJ. Implementing open-access scheduling in an academic practice. Fam Pract Manag. 2006;13(3):59–64. 16568598

[51] Cameron S, Sadler L, Lawson B (2010). Adoption of open-access scheduling in an academic family practice. Can Fam Physician.

[52] Raudenbush SW, Bryk AS (2002). Hierarchical linear models: applications and data analysis methods.

[53] Kim H-J, Fay MP, Feuer EJ, Midthune DN (2000). Permutation tests for joinpoint regression with applications to cancer rates. Stat Med.

[54] Attride-Stirling J (2001). Thematic networks: an analytic tool for qualitative research. Qual Res.

[55] Lavrakas PJ (2013). Presidential address: applying a total error perspective for improving research quality in the social, behavioral, and marketing sciences. Public Opin Q.

[56] Canadian Institute for Health Information (2017). How Canada Compares: Results From The Commonwealth Fund’s 2016 International Health Policy Survey of Adults in 11 Countries — Accessible Report.

